# Subclinical Myocardial Dysfunction and Cardiac Autonomic Dysregulation Are Closely Associated in Obese Children and Adolescents: The Potential Role of Insulin Resistance

**DOI:** 10.1371/journal.pone.0123916

**Published:** 2015-04-23

**Authors:** Domenico Cozzolino, Anna Grandone, Antonio Cittadini, Giuseppe Palmiero, Giovanni Esposito, Annamaria De Bellis, Raffaello Furlan, Silverio Perrotta, Laura Perrone, Daniele Torella, Emanuele Miraglia del Giudice

**Affiliations:** 1 Department of Internal Medicine, Second University of Naples, Naples, Italy; 2 Department of Women, Child and General and Specialized Surgery, Second University of Naples, Naples, Italy; 3 Department of Translational Medical Sciences, Federico II University, Naples, Italy; 4 Department of Advanced Biomedical Sciences, Federico II University, Naples, Italy; 5 Department of Cardiothoracic and Respiratory Sciences, Second University of Naples, Naples, Italy; 6 Internal Medicine, Humanitas Research Hospital, University of Milan, Rozzano, Italy; 7 Department of Medical and Surgical Sciences, Magna Graecia University, Catanzaro, Italy; Niigata University Graduate School of Medical and Dental Sciences, JAPAN

## Abstract

**Background:**

The prevalence of obesity is increasing among children/adolescents. Subtle cardiovascular abnormalities, responsible for a higher mortality later in life, have been reported in obese children/adolescents. The aims of the study were to evaluate cardiovascular autonomic regulation, by means of spectrum analysis of R-R interval variability, and myocardial function, by means of standard and tissue Doppler echocardiography, in a group of non-hypertensive asymptomatic obese children and adolescents; furthermore, the influence of insulin resistance was tested.

**Subjects and Methods:**

R-R interval variability was analyzed during both the 70° head-up tilt and 24-hour electrocardiographic holter monitoring. Spectrum analysis of R-R interval variability provided the indices of sympathetic (low frequency [LF_RR_]) and vagal (high frequency [HF_RR_]) modulation of the sinoatrial node. Homeostasis model assessment of insulin resistance (HOMA-IR) was used to classify obese children/adolescents (n=72) as insulin resistant (n=37) and non-insulin resistant (n=35).

**Results:**

In obese subjects: a) left ventricular mass was significantly (p<0.05) increased, whereas both the e/a ratio and the e'/a' ratio were decreased; b) at rest, HF_RR_ was lower, and the LF_RR_/HF_RR_ ratio was higher; c) during tilting, magnitude of tilt-induced inhibition of HF_RR_ was lower; d) during 24-hour electrocardiographic holter monitoring, LF_RR_ and the LF_RR_/HF_RR_ ratio were higher, whereas HF_RR_ was lower; e) HOMA-IR inversely correlated with both the e'/a' ratio (*r*=-0.655; p<0.001) and the tilt-induced LF_RR_/HF_RR_ ratio (*r*=-0.933; p<0.001); and, f) the e'/a' ratio correlated with the tilt-induced LF_RR_/HF_RR_ ratio (*r*=0.501; p<0.001). Moreover, HF_RR_ at rest, magnitude of tilt-induced HF_RR_ reduction, and the e'/a' ratio in insulin resistant obese children/adolescents were markedly lower when compared with the remaining subjects.

**Conclusions:**

Subclinical abnormalities of myocardial function and of cardiac autonomic regulation were closely associated in obese children/adolescents and both correlated with the degree of insulin resistance.

## Introduction

The pathogenesis of obesity is complex: inheritance, endocrine disorders (including abnormalities of β-cell responsiveness to pituitary), and an incorrect lifestyle have been reported to be implicated in weight gain in humans [1]. The prevalence of obesity is increasing among children and adolescents in western and developing countries [2]. Actually, obesity in childhood is regarded as a major public health problem.

To date, subclinical changes in myocardial function and endothelial regulation, and tendency to hypertension have been reported among obese children and adolescents [3–6]. In addition, autonomic nervous modulation of the sinoatrial node both at rest and during some dynamic conditions, such as tilting and eating, has been found to be disturbed in obese children/adolescents, and a relationship with some metabolic disorders, including β-cell reactivity and insulin resistance, was reported [7–11]. Accordingly, a previous work described a blunted sympathovagal response to tilting in insulin dependent diabetic patients [12]. Moreover, we recently found marked disorders in cardiac autonomic control after eating in obese children/adolescents and a key role for insulin resistance was suggested [10].

Noteworthy, a strong relationship between obesity and insulin resistance exists [13]; however, not all obese subjects are insulin resistant. To date, it is not completely clarified whether cardiac disorders described in overweight subjects, such as changes in myocardial function, chamber geometry, and autonomic regulation of the sinoatrial node, are dependent on obesity *per se*, obesity-related insulin resistance or other factors. A previous study evaluating systolic myocardial function in obese children concluded that some functional abnormalities of the left ventricle (LV) correlated with body mass index (BMI), whereas other LV disorders correlated with insulin resistance and not with BMI [6]. In our opinion, the adverse effects of insulin resistance on cardiovascular function and structure have been extensively studied in obese adults but are not fully elucidated in obese children/adolescents.

Until now, myocardial changes and cardiac autonomic abnormalities have been mostly described separately in obese children/adolescents, and, to present, it is unclear whether such disorders are associated or not in these subjects and whether a common driving force exists. Our hypothesis was that insulin resistance could possibly represent a link between subclinical myocardial dysfunction and cardiac autonomic dysregulation in children/adolescents with obesity.

The aims of the present study were to investigate myocardial function and cardiovascular autonomic regulation in response to tilting in a group of asymptomatic non-hypertensive obese children/adolescents and to evaluate any eventual correlation with insulin resistance. Furthermore, we tested a possible association between myocardial dysfunction and cardiac autonomic dysregulation.

## Subjects and Methods

### Subjects

Between January 2010 and May 2013, 89 obese children and adolescents aged 10 to 16 years were consecutively admitted to the Division of Pediatrics at the Second University of Naples, Italy. A total of 17 obese children/adolescents were excluded because they had one or more of the following exclusion criteria: syncope, hypertension, obstructive sleep apnoea, hereditary, endocrine, and systemic inflammatory diseases, and impaired glucose tolerance. In the remaining 72 subjects and 34 normal-weight controls, clinical examination and blood sampling were performed. The homeostasis model assessment of insulin resistance (HOMA-IR) was calculated as follows: fasting insulin (pmol/liter) x fasting glucose (mmol/liter)/22.5. According to cut points adopted for young people from a Mediterranean area, values of HOMA-IR above 2.67 and 2.22 for pre-pubertal boys and girls, respectively, and above 5.22 and 3.82 for pubertal boys and girls, respectively, indicated a condition of insulin resistance [14]. Based on HOMA-IR values, obese children/adolescents were categorized into two subgroups: insulin resistant (IR+; n = 37) and non-insulin resistant (IR-; n = 35). Obesity was defined as BMI >95th percentile for sex and age, and the smooth L curve, mean, and coefficient of variation method was used to calculate standard deviations scores for BMI (BMI Z-score) [15]. Tanner criteria were used to assess pubertal stage [16]. The Ethics Committee of the Second University of Naples, considering that all the procedures utilized in the study were in accordance with the Helsinki Declaration of 1975 revised in 1983, specifically approved this study. Written informed consent to participate in the study was obtained by parents, caretakers, or guardians on behalf of the minors/children and, where appropriate, by children and adolescents.

### 70° Head-up tilt

After an overnight fast, all children/adolescents underwent the 70° head-up tilt as already described [12]. Briefly, each subject was positioned in supine position on an electrically moved tilt-table while electrocardiogram, heart rate and blood pressure were continuously and non-invasively monitored by a beat-to-beat monitor (CC-NEXFIN, BMEYE NA) throughout entire study protocol. Data acquisition initiated at least 30 minutes after instrumentation. Subsequently, after 5 minutes (baseline), each subject was tilted until 70° head-up position was reached and there remained for 10 minutes.

### 24-Hour electrocardiographic holter monitoring

In the same occasion, 23 IR+, 21 IR-, and 21 control subjects underwent 24-hour ambulatory electrocardiographic recordings with PRIMA-holter (Cardioline, Milano, Italy), with a sampling frequency ranging between 125 and 1000 Hz. All subjects were invited to adhere to their normal course of daily activity.

### Standard and tissue Doppler imaging (TDI) echocardiography

Thirty-five IR+, 33 IR- and 30 control children/adolescents underwent standard echocardiography according to the standards recommended [17] by using an ultrasound mechanical system equipped with a 2.5–3.5 MHz transducer (HD11 XE Philips Medical Systems, Nederland). By means of PW Doppler, e and a waves (early and late peak diastolic velocity, respectively), and the e/a ratio (a composite index of diastolic function) were measured. LV mass was determined according to Devereux’s formula and was indexed to height to the power of 2.7 [18]. Ejection fraction was computed from apical two- and four-chamber views, using the modified Simpson biplane method.

In the same subjects, myocardial velocities were measured with TDI technique. From standard apical view, a sample volume (2 mm) was placed at the level of septal area of mitral annulus, in order to analyze longitudinal tissue motion of LV. The PW tissue Doppler measures obtained were: e' and a' waves (early and late peak diastolic annular velocity, respectively), and s' wave (peak systolic velocity) [19]. The e'/a' ratio was calculated, and a value <1 was considered abnormal. LV filling pressures were approximated from the e/e’ ratio. Much care was taken to have minimal angulation between the ultrasound beam and the plane of cardiac motion.

All echocardiographic measurements were calculated from an average of five consecutive cardiac cycles. All images were stored and analyzed by two experienced cardiologists blinded to the subjects' clinical details.

### Laboratory assays

Blood glucose, insulin, triglycerides, total cholesterol, HDL-cholesterol, and LDL-cholesterol were measured as previously described [10], whereas high sensitivity C-reactive protein (hsCRP) was measured with an ELISA method.

### Cardiovascular autonomic analysis

As already described [10], electrocardiogram and blood pressure were continuously monitored during the 70° head-up tilt for analysis of heart rate and of systolic blood pressure (SBP) variability. Noteworthy, power spectrum analysis of R-R interval variability provides 2 major spectral components: the high frequency (HF_RR_; ~0.25 Hz at rest) and the low frequency (LF_RR_; ~0.10 Hz)), which, when expressed in normalized units (nu), mainly reflect sympathetic and vagal modulation of the sinoatrial node, respectively, and its changes [20]. The LF_RR_/HF_RR_ ratio, a non-dimensional index, was also calculated to assess the reciprocal changes of sympathetic and vagal modulation of the sinoatrial node. The LF oscillatory component of SBP variability (LF_SBP_; ~0.10 Hz), a recognized index of sympathetic modulation of vasomotor activity, was expressed in absolute values [20]. The α index, a marker of arterial baroreceptor sensitivity, was measured as already described [10]. Mean values of these indices obtained during rest period (baseline) were compared with those of the last 5 minutes during tilting.

Power spectrum analysis of heart rate during 24-h holter monitoring was obtained by means of a dedicated software of the same system. In the present study the following frequency-domain indices were measured: HF_RR_ (~0.25 Hz) and LF_RR_ (~0.10 Hz) components, and the LF/HF ratio. Such spectral components were reported as absolute units and as nu.

### Statistical analysis

Statistical analyses were performed with SPSS version 20.0 (SPSS Inc. Chicago, IL, USA). The changes from baseline (Δ values) were calculated as the measurement at peak during tilting minus baseline. Student *t* test for unpaired data was used to compare differences between groups. Not Gaussian distributed variables (e.g.; BMI Z-score) were analyzed by means of Kruskall-Wallis test. Two-way ANOVA for repeated measures was used to test for differences within-group. Categorical variables were presented as percentages (%), and the significance of differences was examined with the χ^2^ test. The Pearson’s *r* was used to assess the correlations between the clinical and metabolic factors and the e'/a' ratio and tilt-induced Δ changes in the LF_RR_/HF_RR_ ratio. In addition, the clinical and metabolic factors independently related to the e'/a' ratio and tilt-induced Δ changes in the LF_RR_/HF_RR_ ratio were established through multiple stepwise regression analysis (with stepping method criteria: probability of F to enter ≤ 0.05 and to remove ≥ 0.10). Moreover, the Pearson’s *r* calculation was also used to evaluate the individual association between the e'/a' ratio and tilt-induced Δ changes in the LF_RR_/HF_RR_ ratio. Data were expressed as mean±SD. All statistical tests were two-sided. Significance was considered when p<0.05.

## Results

hsCRP and LDL-cholesterol levels in obese children/adolescents were significantly higher (p<0.001) than in referents, whereas HDL-cholesterol was lower (p<0.001) (Table 1).

**Table 1:** Clinical characteristics of insulin resistant and non-insulin resistant obese and control children/adolescents.

	IR + (n = 37)	IR- (n = 35)	Controls (n = 34)
Sex (M/F)	17/20	15/20	17/17
Age (yr)	12.9±2.1	12.7±2.1	12.6±2.0
Weight (Kg)	86±21*	85±21*	47±7
Body mass index (Kg/m^2^)	33±10*	33±9*	24±1
Body mass index Z-score	2.7±0.7*	2.6±0.7*	0.7±0.6
Body surface area (m^2^)	1.5±0.7	1.4±0.7	1.2±0.5
Fasting glucose (mg/dl)	87.1±8.4* †	74.4±6.5	75.7±4.4
Fasting insulin (μU/ml)	17.1±6.3* †	7.7±4.4	6.7±3.0
HOMA-IR	3.7±0.7* †	1.4±0.7	1.3±0.4
hsCRP (mg/dl)	0.67±0.14* †	0.55±0.11*	0.40±0.14
Total cholesterol (mg/dl)	144±17	145±16	143±16
HDL-cholesterol (mg/dl)	41±7*	43±7*	54±6
LDL-cholesterol (mg/dl)	82±16*	81±17*	70±15
Triglycerides (mg/dl)	107±44	105±43	97±25
Systolic blood pressure (mm Hg)	120±10*	121±7*	107±6
Diastolic blood pressure (mm Hg)	66±7	67±7	66±6
Heart rate (beats/min)	71±10	70±10	69±10
LF_RR_ (ms^2^)	767±94* †	651±105	645±98
LF_RR_ (nu)	49±4* †	43±7	42±7
HF_RR_ (ms^2^)	420±111* †	551±114*	690±110
HF_RR_ (nu)	30±7* †	37±7*	44±5
LF_RR_/HF_RR_	2.1±0.7* †	1.2±0.4*	1.0±0.1
LF_SBP_ (mm Hg^2^)	3.5±0.7*	3.5±0.6*	2.4±0.6
α Index (ms/mm Hg)	15.0±0.9*	14.4±1.1*	17.6±1.1

Data represent mean±SD;

*, p<0.05 vs controls;

^†^, p<0.05 vs IR-

IR+, insulin resistant obese subjects; IR-, non-insulin resistant obese subjects; HOMA-IR, homeostasis model assessment of insulin resistance; hsCRP, high sensitivity C-reactive protein; LF_RR_, low frequency power of R-R variability; nu, normalized units; HF_RR_, high frequency power of R-R variability; LF_SBP_, low frequency power of systolic blood pressure variability.

### Standard and TDI echocardiography

By standard echocardiography, LV diameters, thickness, and mass, and left atrium in obese subjects were found significantly greater (p = 0.002, at least) than in controls, whereas e wave and the e/a ratio were lower (p = 0.002, at least) (Table 2). LV end-systolic diameter and end-diastolic thickness of posterior wall in IR+ subjects were greater (p = 0.02, at least) compared with IR- individuals (Table 2). The e/a ratio was inverted in 3/35 IR+ subjects and in none of the remaining subjects (p = n.s.).

**Table 2:** Standard and tissue Doppler echocardiographic measures of left chambers in insulin resistant and non-insulin resistant obese and control children/adolescents.

	IR + (n = 35)	IR- (n = 33)	Controls (n = 30)
Ventricular end-diastolic diameter (mm)	45.6±1.7*	45.6±2.3*	39.9±4.3
Ventricular end-systolic diameter (mm)	29.4±2.1* †	27.6±1.5*	25.5±3.1
Ejection fraction (%)	61.4±1.3	61.8±1.7	62.0±1.9
Interventricular septum dimension (mm)	8.7±0.9*	8.4±0.8*	7.2±0.9
Posterior wall dimension (mm)	8.7±0.6* †	8.3±0.7*	7.1±0.8
Ventricular mass/h^2,7^ (g/m^2,7^)	37.7±5.8*	37.8±5.2*	26.2±4.4
Atrial dimension (mm)	34.3±1.2*	33.5±2.0*	27.7±4.0
e (cm/s)	95.1±15.8*	95.9±13.6*	104.2±12.9
a (cm/s)	58.5±9.6	55.7±8.8	57.9±12.3
e/a	1.7±0.3*	1.6±0.4*	1.9±0.3
s’ (cm/s)	8.4±0.7	9.1±2.7	9.2±2.2
e’ (cm/s)	13.2±2.8	13.4±2.2	14.4±3.2
a’ (cm/s)	6.9±1.7	6.6±1.7	6.7±1.4
e’/a’	1.9±0.4* †	2.1±0.4*	2.3±0.3
e/e’	7.2±1.6	7.2±1.5	7.3±2.0

Data represent mean±SD;

*, p<0.05 vs controls;

^†^, p<0.05 vs IR-

IR+, insulin resistant obese subjects; IR-, non-insulin resistant obese subjects; h, eight; e, early peak diastolic velocity; a, late peak diastolic velocity; s', peak systolic tissue Doppler velocity; e’, early peak diastolic tissue Doppler velocity; a’, late peak diastolic tissue Doppler velocity.

By TDI echocardiography, the e'/a' ratio in obese subjects was found significantly (p<0.03, at least) lower than in referents (Table 2). By comparing the subgroups, the e’/a’ ratio in IR+ subjects was lower (p<0.05) than in IR- counterparts (Table 2).

### Cardiovascular autonomic analysis

#### Baseline

At rest, SBP, the LF_RR_/HF_RR_ ratio and LF_SBP_ in obese children/adolescents were greater (p = 0.006, at least) than in referents, whereas HF_RR_ and the α index were lower (p<0.001) (Table 1). Furthermore, LF_RR_ and the LF_RR_/HF_RR_ ratio in IR+ subjects were higher (p<0.001) than in IR- individuals, whereas HF_RR_ was lower (p<0.001) (Table 1).

#### 70° Head-up tilt

**Fig 1:**
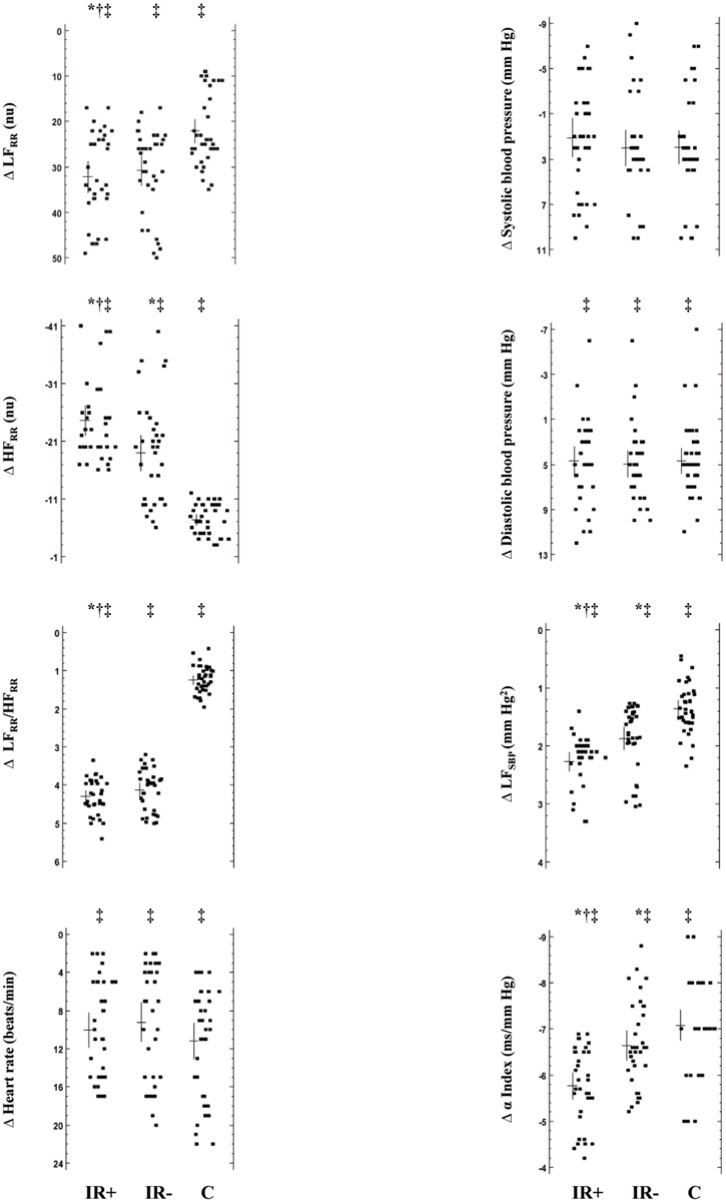
Δ Changes induced by tilting on hemodynamics and cardiovascular autonomic regulation in insulin resistant and non-insulin resistant obese and control children/adolescents. LF_RR_, low frequency power of R-R variability; nu, normalized units; HF_RR_, high frequency power of R-R variability; LF_SBP_, low frequency power of systolic blood pressure variability; IR+, insulin resistant obese subjects; IR-, non-insulin resistant obese subjects; C, control subjects. Individual data in the 3 groups of subjects together with their mean value and relative SD are depicted; tilt-induced Δ changes were compared; *, p<0.05 vs controls; †, p<0.05 vs IR-; ‡, p<0.05 vs baseline.

Compared with baseline, diastolic blood pressure, LF_RR_, the LF_RR_/HF_RR_ ratio, heart rate, and LF_SBP_ significantly (p = 0.007, at least) rose during tilting in all subjects, whereas HF_RR_ and the α index decreased (p = 0.002, at least), as expected (Fig 1). Tilt-induced Δ changes in HF_RR_ component and LF_SBP_ in obese subjects were significantly (p<0.02, at least) lower than in referents, whereas decrement in the α index was greater (p = 0.004, at least) (Fig 1). When tilted, magnitude of Δ changes in LF_RR_, HF_RR_, the LF_RR_/HF_RR_ ratio, and LF_SBP_ in IR+ subjects was significantly (p = 0.02, at least) lower compared with IR- individuals, whereas the α index decreased greatly (p<0.004) (Fig 1).

#### 24-Hour electrocardiographic holter monitoring

In obese subjects, LF_RR_ component (795±76 ms^2^, 50±7 nu) and the LF_RR_/HF_RR_ ratio (1.5±0.5) were significantly (p<0.01, at least) higher than in control counterparts (642±99 ms^2^, 43±7 nu, and 1.0±0.2, respectively), whereas HF_RR_ component was lower (499±105 vs 673±116 ms^2^; 32±8 vs 42±7 nu). Conversely, LF_RR_ (803±67 ms^2^, 51±7 nu) and HF_RR_ (488±116 ms^2^, 31±9 nu) components and the LF_RR_/HF_RR_ ratio (1.6±0.7) in IR+ subjects were found not significantly different when compared with those in IR- subjects (LF_RR_: 787±85 ms^2^, 49±8 nu; HF_RR_: 509±94 ms^2^, 33±8 nu; the LF_RR_/HF_RR_ ratio: 1.4±0.3).

#### Reproducibility

Intra-observer variability of the echocardiographic parameters was tested by repeating the measurements in 30 randomly selected subjects (15 obese and 15 controls) and on two separate occasions (1–15 days apart). To assess the inter-observer variability, the measurements were performed offline from video recordings by another cardiologist who was unaware of the results of the first examination. Variability was calculated as the mean percentage error, derived from the difference between the two sets of measurements divided by the mean of the observations. Intra-observer and inter-observer variability percentages for the main echocardiographic parameters ranged from 2.5% to 4.9%.

#### Correlations

**Fig 2:**
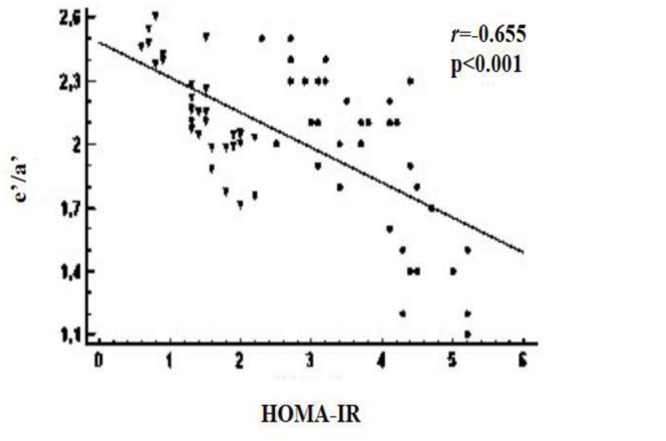
Relationship between the left ventricular e'/a' ratio and HOMA-IR index in the whole group of obese subjects (insulin resistant, circles; non-insulin resistant, triangles). e’, early peak diastolic tissue Doppler velocity; a’, late peak diastolic tissue Doppler velocity; HOMA-IR, homeostasis model assessment of insulin resistance.

**Fig 3:**
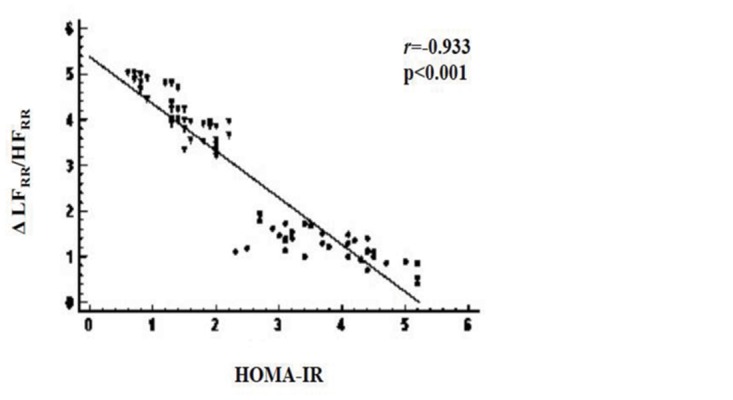
Relationship between tilt-induced Δ changes in the LF_RR_/HF_RR_ ratio and HOMA-IR index in the whole group of obese subjects (insulin resistant, circles; non-insulin resistant, triangles). LF_RR_, low frequency power of R-R variability; HF_RR_, high frequency power of R-R variability; HOMA-IR, homeostasis model assessment of insulin resistance.

The e'/a' ratio significantly and inversely correlated with HOMA-IR (*r* = -0.655; p<0.001) (Fig 2) and hsCRP (*r* = -0.346; p = 0.004) in obese children/adolescents, but not with BMI Z-score (*r* = 0.179; p = 0.143) and the main other variables (age, triglycerides, total cholesterol, HDL-cholesterol, LDL-cholesterol, blood pressure, and HF_RR_ at rest). Similarly, tilt-induced Δ changes in the LF_RR_/HF_RR_ ratio inversely correlated with HOMA-IR (*r* = -0.933; p<0.001) (Fig 3) and hsCRP (*r* = -0.250; p = 0.04) in obese people, but not with BMI Z-score (*r* = -0.115; p = 0.334) and the main other variables. In multiple stepwise regression analysis, HOMA-IR correlated with both the e'/a' ratio (SE = 0.017; coefficient = -0.074; p<0.001) and tilt-induced Δ changes in the LF_RR_/HF_RR_ ratio (SE = 0.060; coefficient = -0.652; p<0.001) in obese subjects. In addition, the e'/a' ratio related with tilt-induced Δ changes in the LF_RR_/HF_RR_ ratio (*r* = 0.501; p<0.001) in obese people.

## Discussion

The negative impact of body fat excess on ventricular function and morphology has been already demonstrated [21, 22]. Such an increased cardiac morbidity in obesity likely depends on many factors, including lipotoxicity, changes in myofilament proteins, some cytokines from epicardial adipose tissue (locally responsible for unfavourable effects on contractile properties of cardiomyocytes and coronary arteries), and insulin resistance [23–25].

Obese children/adolescents of the present study showed subclinical changes in LV diastolic function, especially among IR+ individuals. Accordingly, a recent echocardiographic study based on strain rate analysis reported an impairment of myocardial deformation properties in obese children [6]. In our study, myocardial function has been also investigated by means of TDI support, which is recognized as a validated, sensitive and reproducible tool capable to precisely measure longitudinal myocardial ventricular motion independently on geometric assumptions, endocardial border tracing, and load conditions. Changes in diastolic function closely correlated with hsCRP and HOMA-IR among obese people, thus suggesting a contributing role for insulin resistance, probably due to its property to negatively influence some cardiomyocyte metabolic pathways. To support this hypothesis, data from animal models showed that obesity-related insulin resistance increased fatty acid availability and uptake, caused a shift in myocardial substrate metabolism toward a preference for fatty acid utilization, and, finally, provoked myocardial injury [23]. Accordingly, a previous study conducted in young obese women reported myocardial deficiency and a parallel increase in cardiomyocyte fatty acid uptake, which strictly correlated with insulin resistance [25]. In addition, a previous work from our laboratory described an impaired myocardial response to double stress (exerted by insulin plus exercise) in insulin resistant obese adults, likely secondary to deficiency of insulin sensitive pyruvate dehydrogenase complex [26].

Obese children/adolescents of the present study showed an impaired baroreflex sensitivity, a decreased cardiac vagal tone and an increased sympathetic activity, as indicated by higher values of the LF_RR_/HF_RR_ ratio. Cardiac sympathovagal regulation has been indirectly investigated by heart rate variability analysis during both a long period (24 hours) and stress exerted by gravitational stimulus (tilting). LF_RR_ and HF_RR_, despite their mixed origin [27], when measured in normalized units (or as the LF_RR_/HF_RR_ ratio, which represents their reciprocal relationship), provide validated quantitative markers of cardiac sympathetic and vagal modulation, respectively [12]. Moreover, low frequency oscillations of cardiac sympathetic activity have been found to be strictly correlated with those of muscle sympathetic nerve activity during tilting [28].

During tilting, our obese children/adolescents, especially IR+ subjects, exhibited a blunted enhancement of cardiac sympathetic activity together with a scanty reduction of vagal tone. Moreover, a strong (and inverse) relationship between magnitude of tilt-induced cardiac autonomic response and the degree of insulin resistance (expressed by HOMA-IR index) was found in obese subjects. Similarly, a recent work conducted in obese children/adolescents reported parasympathetic withdrawal and sympathetic hyperactivity at rest together with a disturbed cardiac autonomic regulation after eating, which were particularly pronounced among IR+ subjects [10]. Furthermore, insulin resistant individuals with impaired fasting glucose have previously shown cardiac autonomic dysfunction, and a pathogenic link between increased oxidative stress, low-grade chronic inflammation and cardiac autonomic dysregulation was proposed [29]. The blunted cardiac autonomic response to dynamic challenges (exerted by tilting) in obese subjects of the present study could probably be ascribed to early functional autonomic neuropathy, similar to that reported in diabetic patients [12]. We attempted to explain our findings by hypothesizing that a coexistent condition of chronic low-grade inflammation combined with increased oxidative stress in our obese children/adolescents, particularly pronounced among those with insulin resistance, could have played a contributing role in the development of cardiovascular autonomic dysregulation. Moreover, the subsequent abnormality in the autonomic nervous system caused by inflammation might, in turn, enhance the inflammation itself by the "cholinergic anti-inflammatory pathway": acetylcholine has been suggested to inhibit the production of pro-inflammatory cytokines from macrophages [30]. In support of these hypotheses, our obese subjects presented higher plasma levels of the circulating inflammatory marker hsCRP and lower values of the vagal index HF_RR_, which were markedly abnormal among insulin resistant individuals; in addition, hsCRP inversely correlated with amplitude of sympathovagal response to tilting.

The e'/a' ratio values and magnitude of sympathovagal response to tilting were closely related: we are the first to describe a close association between cardiac autonomic imbalance and myocardial impairment in obese children/adolescents.

Collectively, the findings of the present study suggest that insulin resistance, rather than obesity *per se*, is associated with both a global dysregulation of the autonomic nervous system and subclinical disorders of myocardial function in obese children and adolescents; as previously suggested by other authors, low-grade inflammation and/or enhanced oxidative stress might mediate this association.

### Clinical implications

There is evidence that both subclinical myocardial disorders and cardiac autonomic abnormalities, often early operative in obese children/adolescents, are potentially responsible for an overt global myocardial dysfunction and acute cardiovascular events later in life [31]. Nevertheless, a diet-induced ~4% BMI reduction in obese children has been reported to improve diastolic function [32]. However, further studies are needed to confirm such findings and to clarify whether a proper and prompt lifestyle-based intervention devoted at correcting weight excess (and, likely obesity-related insulin resistance) in obese children/adolescents could limit or prevent (better) the negative impact of fat excess on myocardial function and cardiac autonomic control.

### Study limitations

In order to exclude a condition of undiagnosed hypertension and/or sleep apnoea, each individual would have to be subjected to ambulatory blood pressure monitoring and sleep polysomnography, which were regarded as impractical in the present study. However, all the subjects recruited in the study exhibited: a) normal values of systolic and diastolic blood pressure, both in at least three different occasions, according to current cut points (i.e., <95th percentile for age, sex, and height) [33]; and, b) no habitual snoring and/or apnoea (according to parent recall), both important symptoms of obstructive sleep apnoea. Some influencing factors on heart rate variability, including duration of obesity and physical training, were not precisely known. Finally, usual recognized limitations of some TDI measures and the angle dependence should be considered when evaluating our results.

### Conclusions

In childhood, the negative impact of obesity on myocardial function and on cardiac autonomic control is likely influenced by insulin resistance. Concurrently, subclinical myocardial dysfunction and cardiac autonomic dysregulation are closely associated in obese children/adolescent. Thus, the evaluation of cardiac autonomic regulation could represent an additional tool to assess the cardiovascular impact of weight excess in young obese subjects.

## Supporting Information

S1 File Additional Information. (DOC)
