# Supplementary material for: Subclinical Myocardial Dysfunction and Cardiac Autonomic Dysregulation Are Closely Associated in Obese Children and Adolescents: The Potential Role of Insulin Resistance
Source: PLoS One. 2015 Apr 23;10(4):e0123916. doi: 10.1371/journal.pone.0123916 (PMC4408004; doi:10.1371/journal.pone.0123916)
Supplement: S1 File — (DOC) [file pone.0123916.s001.doc]

**Additional Information:**

**Data availability.** Data are stored at the Department of Woman, Child and General and Specialized Surgery of the Second University of Naples for researchers who meet the criteria for access to confidential data.

Due to ethical restrictions implemented by our Medical Ethics Committee, our data are available upon request to Domenico Cozzolino, Department of Internal Medicine, Second University of Naples, via Pansini 5, 80131 Naples, Italy; e-mail: [domenico.cozzolino@unina2.it](mailto:domenico.cozzolino@unina2.it).

The dataset, named “Cuore ed obesità nell’Infanzia”, is owned by the Department of Woman, Child and General and Specialized Surgery of the Second University of Naples.
